# Predictive Modeling of Molecular Mechanisms in Hydrogen Production and Storage Materials

**DOI:** 10.3390/ma16176050

**Published:** 2023-09-03

**Authors:** Tanumoy Banerjee, Ganesh Balasubramanian

**Affiliations:** Energy Research Center, Lehigh University, 117 ATLSS Drive, Bethlehem, PA 18015, USA

**Keywords:** hydrogen production, storage efficiency, retention rate, porous nanostructures, carbonaceous materials, molecular dynamics, electrolysis

## Abstract

Hydrogen has been widely considered to hold promise for solving challenges associated with the increasing demand for green energy. While many chemical and biochemical processes produce molecular hydrogen as byproducts, electrochemical approaches using water electrolysis are considered to be a predominant method for clean and green hydrogen production. We discuss the current state-of-the-art in molecular hydrogen production and storage and, more significantly, the increasing role of computational modeling in predictively designing and deriving insights for enhancing hydrogen storage efficiency in current and future materials of interest. One of the key takeaways of this review lies in the continued development and implementation of large-scale atomistic simulations to enable the use of designer electrolyzer–electrocatalysts operating under targeted thermophysical conditions for increasing green hydrogen production and improving hydrogen storage in advanced materials, with limited tradeoffs for storage efficiency.

## 1. Introduction

Energy has been categorically classified into renewable and non-renewable forms, with approximately two-thirds of the world’s energy demand being fulfilled by non-renewable liquid and gaseous fossil energy resources (mostly petroleum and natural gas) [1]. The primary detrimental effects of these fossil energy sources are their emission of CO_2_ and other harmful greenhouse gases, causing global warming and climate change. This damaging impact, along with the ever-increasing price of fossil energy and health and safety concerns, is pushing the international community to develop and look for other less harmful and preferable renewable energy sources to meet their daily demands [2,3], including solar, wind, hydroelectricity, tidal, geothermal, and electrochemical routes [2]. However, owing to their unpredictable nature, these renewable energy sources would only be feasible for use with an energy storage system [4,5].

**Figure 1:**
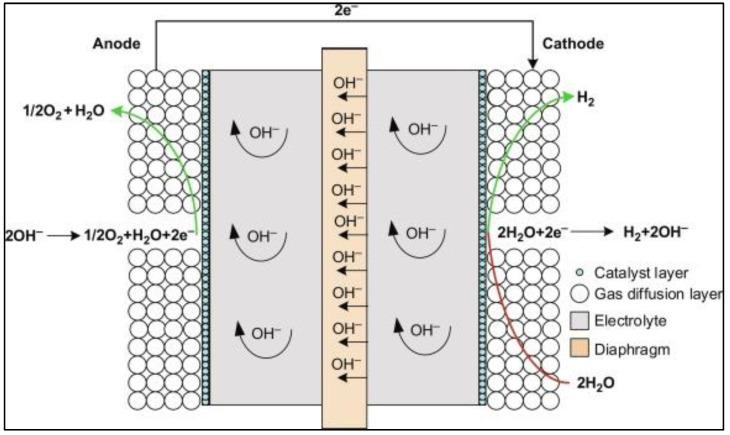
Representative schematic of an alkaline water electrolyzer, where hydrogen is evolved in the cathode. Reprinted with permission from [18].

Currently, among the feasible energy storage technologies, electrochemical energy storage has been considered the most effective and easily convertible. Since stored hydrogen can be readily transformed into electricity using fuel cell technology, the use of electricity derived from renewable energy to electrolyze water and produce hydrogen is one of the efficient electrochemical energy storage technologies, which has led to the development of effective water electrolysis technologies for hydrogen production in the gaseous form [6,7]. Also, the potential of hydrogen as a substitute for gasoline, diesel, and biofuels in the automotive and fuel cell industry holds promise. A recent study showed that various factors, such as energy expenses and process efficiency, significantly influence the potential for cost-effective hydrogen generation via electrolysis [6]. Currently, 48% of the total hydrogen in the world is produced from natural gas: 30% from oils, 18% from coal, and only 4% from electrolysis. Additionally, 95% of all hydrogen is produced via steam methane reforming, while the remaining hydrogen evolution processes (gasification of coal, hydrogen from biomass, and water electrolysis) constitute only ~5% [8,9,10,11]. Water electrolysis has been proven to be the best method for hydrogen production, given its zero-carbon emission, and it only uses renewable H_2_O, with the byproducts being pure hydrogen and oxygen. However, the hydrogen conversion efficiency for this process is lower (40~60%) than steam methane reforming (SMR), which is the most developed contemporary hydrogen production technology [2,12]. Depending on the operating temperature and the type of electrolytes and electrodes used, hydrogen evolution reactions can be further classified into three different electrolysis technologies: alkaline water electrolysis (40~100 °C), proton exchange membrane electrolysis (20~100 °C), and solid oxide electrolysis (500~1000 °C) technologies [12,13,14]. Alkaline water electrolysis (as illustrated in Figure 1) is currently the most developed technology for hydrogen production via electrolysis; however, it has certain limitations, including a lower current density and operating pressure that make it energy inefficient. On the other hand, solid oxide electrolysis technology, operating at a higher temperature, is an immature yet growing technology with higher hydrogen production rates [15,16,17].

**Figure 2:**
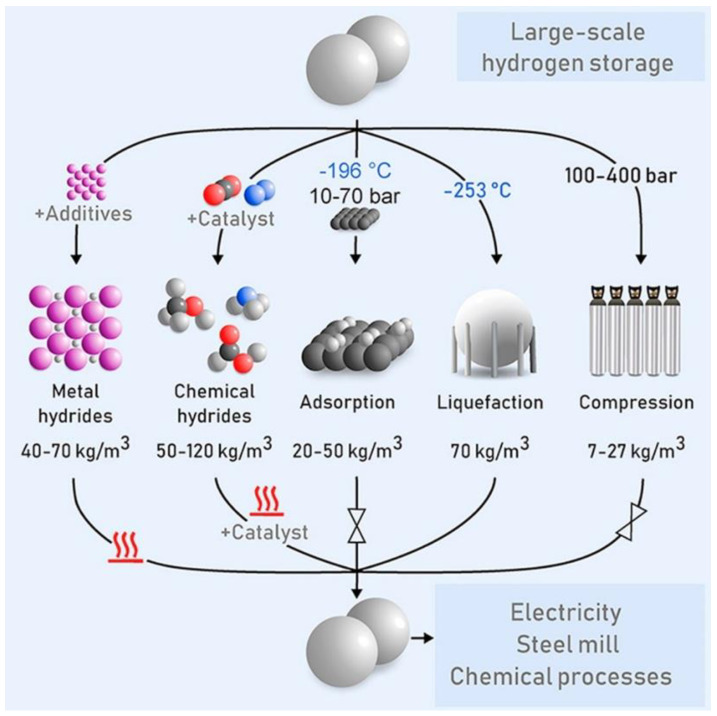
The different types of technologies available for hydrogen storage at a large scale and over a wide range of operating conditions. Reprinted with permission from [19].

In parallel, a very high heating value and a very low mass density render the storage of hydrogen to be one of the major technical challenges. This storage technology mainly consists of three categories: physical storage (hydrogen is stored either in gaseous or liquid form at a very high pressure), physical adsorption (atomic or molecular hydrogen creates weak van der Waals bonds with specific porous materials and is adsorbed into the walls and inside the porous structure), and chemical absorption (atomic and molecular hydrogen is chemically absorbed into metal and chemical hydrides), as illustrated in Figure 2 [19]. However, these technologies are challenged for storing large quantities of hydrogen for a long period. Considering the significant potential of hydrogen as a future energy source and its notable prospects for energy systems, the imminent questions that arise include how we can develop a comprehensive way to generate hydrogen more efficiently and how we can store it effectively without compromising the production rate and energy losses during storage to increase the hydrogen economy. With the goal of understanding the challenges and associated scientific innovations, we provide a critical literature review on the state-of-the-art computational approaches applied for advanced materials in hydrogen production from electrolysis and its storage. Multiscale molecular simulations in predicting the hydrogen production rate, production efficiency, and efficacy with different materials for storage are advantageous to limit and accelerate experimental measurements. On the other hand, conventional experimental techniques to perform hydrogen evolution reactions (HERs) from electrolysis and examination of gaseous hydrogen interactions with various metal alloys and carbonaceous materials can provide reliable results but are resource-expensive and relatively sluggish relative to the rapid computational approaches.

## 2. Hydrogen Production and Storage

Electrocatalysts are necessary to accelerate hydrogen production using water electrolysis since the oxidation–reduction reaction of water that produces pure H_2_ at the cathode and pure O_2_ at the anode is typically sluggish. The two half-reactions that occur during the electrochemical splitting of water include the oxygen evolution reaction (OER) on the anode and hydrogen evolution reaction (HER) on the cathode; so, the overall electrolysis reaction can be expressed as $2H_{2}O\to 2H_{2}+O_{2}$. This reaction happens under a standard cell voltage of 1.23 V operating in a standard thermodynamic condition of 1 atm pressure and 25 °C temperature [2]. While electrocatalysts help to increase the reaction kinetics of the OER and HER, they are not as widely used for alkaline water electrolysis as in the case of the proton exchange membrane and solid oxide electrolysis [2]. Several noble metal catalysts like Ru, Ir, and Pt have been used for their small overpotential, but because they are essentially expensive and also increase the hydrogen production cost, metal-based nanoparticles like Ag, Ru, RuO_2_, and IrO_2_ are gaining importance for their ability to dissociate water quickly with an enhanced electron transfer rate [19,20,21]. One such example is demonstrated by Li et al., where a very small overpotential of only 281 mV with a current density of 100 A/m^2^ is achieved by coating a layer of discontinuous IrO_2_ on the RuO_2_ surface [22]. Irrespective of the high catalytic efficiency for the OER, the expensive noble metal compounds are being replaced by nickel-based oxides, hydroxides, double hydroxides, phosphides (NiO-based films, Fe-doped NiO_x_, NiFe-layered double hydroxides, Fe-doped Ni_2_P, FeNiP, etc.), Ni-based alloys (NiCo, NiMo, NiCoCr, NiCoMn, etc.), and cobalt and manganese-oxides (Co_3_O_4_, Fe-Co_3_O_4_, MnCo_2_O_4_, MnO_2_, Ni with Mn_2_O_3,_ etc.) owing to their low overpotentials and ability to dissociate water at higher current density [23,24,25,26,27,28,29]. Generally, it is found that the ability to perform as an electrocatalyst for three common metals (Ni, Fe, and Co) follows the order Fe < Co < Ni [30]. Several research directions emerge, including the development of new and cost-effective production methods for metal-based catalysts having high electrocatalytic performances, the selection of new catalysts based on the fundamental mechanisms during the HER and OER, and the design of non-noble metal-based catalysts for the HER and OER in proton exchange membrane (PEM) electrolysis and solid oxide electrolysis cells (SOECs) [2]. As for alkaline water electrolysis, generating a larger amount of hydrogen production yield requires a higher net electricity input into the electrolyzer stack. This net demand in electricity for hydrogen production is determined by the electrochemistry of hydrolysis and the electrochemical model of the water dissociation process. Some of these models have been previously developed and are used to successfully predict the electrochemical behavior of the alkaline water electrolyzer stack under different pressure and temperature ranges [31,32]. The model developed by Ulleberg [31] has been improved by Sánchez et al. [32] to incorporate the polarization curve model to derive the final optimized voltage for each electrolyzer cell ${(V}_{cell})$:

$$V_{cell}=V_{rev}+\left[\left(r_{1}+d_{1}\right)+r_{2}\cdot T+d_{2}\cdot p\right]+s\cdot \log\left[\left(t_{1}+\frac{t_{2}}{T}+\frac{t_{3}}{T^{2}}\right)\cdot i+1\right]$$

$V_{rev}$ is the reversible cell voltage = 1.23 V for water dissociation at standard conditions (25 °C and 1 atm pressure).

For hydrogen storage, typically, two contemporary approaches are considered: (a) physical storage of hydrogen as compressed gas and cryogenic liquid and (b) material-based or solid-state hydrogen storage. Currently, most of the hydrogen worldwide is being stored in compressed gaseous forms at a pressure of 350–700 bars as it is a simple technology with a fast filling-releasing rate. However, the major drawback is that the volumetric density of hydrogen does not rise with increasing pressure, posing a critical constraint on storage tank design [33]. To compensate for some of these challenges, cryogenic hydrogen storage has been incorporated, but the large energy consumption for hydrogen liquefication is the primary concern. Also, the storage efficiency is reduced with time due to continuous heat input into the storage tank, which leads to the evaporation of significant amounts of hydrogen [33,34]. In the case of material-based storage, its high efficiency is correlated to the large amount of hydrogen that can be stored at ambient conditions within a small volume, and this mainly constitutes two processes, viz., absorption and adsorption. In case of absorption, hydrogen atoms react and integrate into the lattice structure of Li, Mg, Na, Ti, and other similar metals (M) to form metal hydrides ($MH_{x}$) following the generalized chemical reaction, $M\left(s\right)+\frac{x}{2}H_{2}\left(g\right)↔MH_{x}\left(s\right)+Q$, where Q refers to the heat of the formation of the hydride. On the other hand, hydrogen adsorption is a surface-level interaction happening at low pressure and occurs predominantly with a porous material having a large surface area-to-volume ratio (generally graphite, carbon nanotubes, boron nitride nanotubes, and C_60_ buckyballs) because this increases the rate of hydrogen kinetics and reduces the binding energy [35]. However, the major disadvantage is the relatively low hydrogen storage capacity (in %wt) and a low gravimetric density of hydrogen.

## 3. Computational Models of Molecular Hydrogen Production

Most of the large-scale hydrogen production happens using steam methane reforming. Cassone et al. performed a first-of-its-kind ab initio molecular dynamics (AIMD) simulation for hydrogen production from neat ethanol at room temperature (300 K) without the presence of any catalysts by applying an external static electric field from 0 V/Å to 0.6 V/Å with a step increment of 0.05 V/Å [36]. Since this analysis is performed with two different systems, viz., (a) anhydrous ethanol and (b) aqueous solution of ethanol with 50% water, the results suggest that both have a specific dissociation threshold (0.25 V/Å for ethanol–water mixture and 0.3 V/Å for anhydrous ethanol) and the average lifetimes of static ions and ionic wires increase rapidly at field strength > 0.4 V/Å only for the ethanol–water mixture.

**Figure 3:**
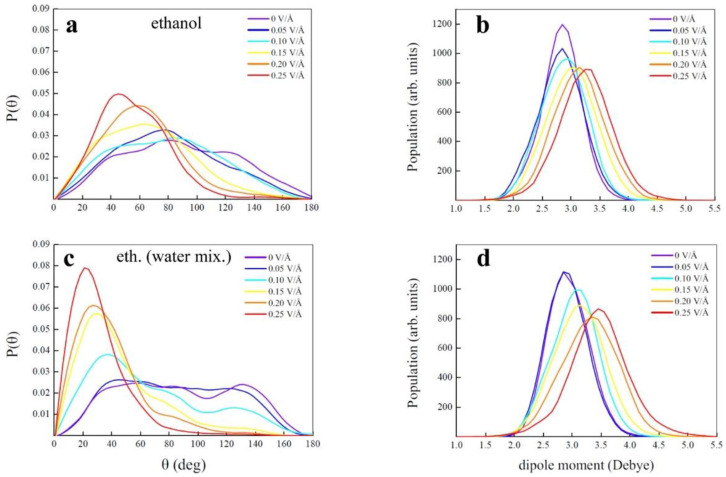
(**a**,**c**) Distribution of the θ angle formed between the vector identifying the O-H bonds of the ethanol molecules and the field directions in (**a**) neat ethanol and (**c**) ethanol–water mixture from zero field regime up to 0.25 V/Å. (**b**,**d**) Dipole moment distribution functions of the ethanol molecules in (**b**) neat ethanol and (**d**) ethanol aqueous solution. Reprinted with permission from [36].

Figure 3 reproduces the O-H bond angle under the application of electric field potential and corresponding dipole moment for both the anhydrous ethanol and ethanol–water mixture. Here, the O-H bonds align themselves congruently with the applied electric field for the ethanol–water mixture relative to the anhydrous ethanol case. But, only anhydrous ethanol is able to produce hydrogen by the application of an external electric field, while aqueous ethanol cannot because of the highly percolated H bond reaction. In other words, at high electric fields, the ethanol–water mixture enhances its entropy by sustaining ionic conduction along an H bond network, but anhydrous ethanol increases its entropy by producing hydrogen molecules, and the same amount of acetaldehyde $\left(CH_{3}CHO\right)$. The process of releasing hydrogen from anhydrous ethanol is triggered by the recombination of the hydride (H^−^) and proton (H^+^) with acetaldehyde and an ethanol molecule as byproducts, as follows:

$$2CH_{3}CH_{2}OH\overset{0.30\frac{V}{Å}}{\to }CH_{3}CH_{2}OH_{2}^{+}+CH_{3}CH_{2}O^{-}$$

$$2CH_{3}CH_{2}OH\overset{0.55\frac{V}{Å}}{\to }H_{2}+CH_{3}CHO+CH_{3}CH_{2}OH$$

**Figure 4:**
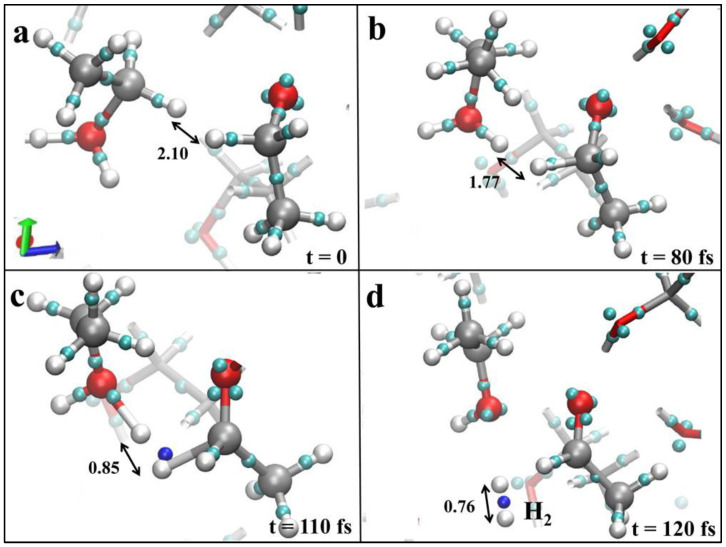
(**a**–**d**) The hydrogen evolution reaction mechanism from liquid ethanol in presence of static unidirectional (along +*z*-axis) electric field strength of 0.55 V/Å. White, silver, and red refer to hydrogen, carbon, and oxygen atoms, respectively, and cyan is the charge center. Formation of electron pair is denoted in blue at 110 fs in (**c**), and the formation of H_2_ is shown at 120 fs in (**d**). All the distance dimensions are in Å. Reprinted with permission from [36].

The decomposition of ethanol happens at or above a field strength of 0.30 V/Å, and the formation of hydrogen is observed when the field strength increases to 0.55 V/Å, as given in Figure 4a–d [36]. Interestingly, all these reactions have been carried out at room temperature and without the presence of any chemical or surface catalysts like sulfuric acid or any Ni-, Pt-, or Au-based electrocatalytic surfaces.

**Figure 5:**
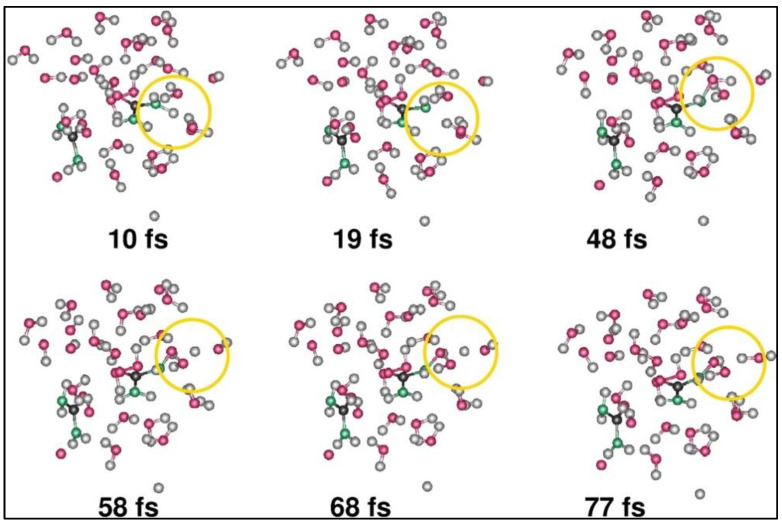
Electrochemical reaction of urea at the starting phases of molecular dynamics simulation where the oxidation of one of the urea molecules and the formation of N-O bond is observed. The yellow circle represents the location where urea molecule gets oxidized in two stages with two OH ˙ radicals, ultimately forming a N-O bond. Here, the N, C, O, and H atoms are in green, black, pink, and gray, respectively. Reprinted with permission from [38].

**Figure 6:**
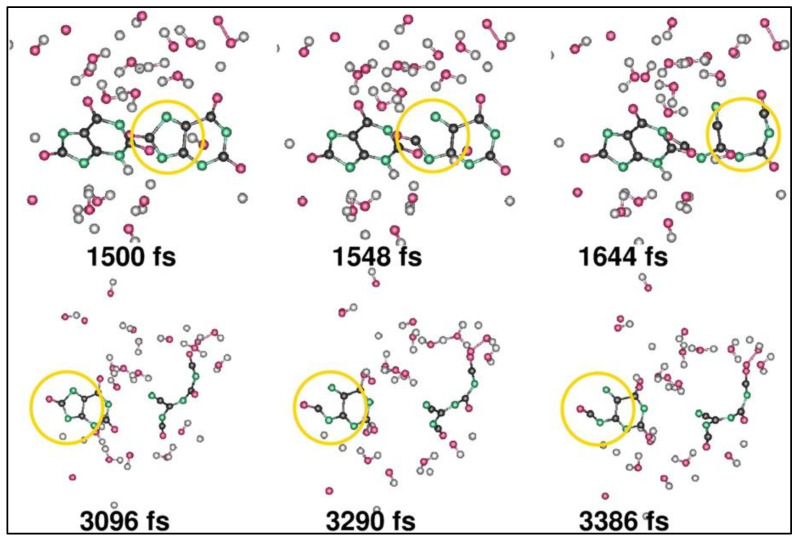
The ring opening is replicated where one of the ring systems of uric acid during its anodic half-reaction is completely opened following the removal of hydrogen atoms (shown by the yellow circles in the top 3 snapshots), while only one side of the ring is broken for the other ring system (shown by the yellow circles on the bottom 3 snapshots) of urea chain during the anodic half-reaction. Reprinted with permission from [38].

Car–Parrinello Molecular Dynamics (CPMD) simulation [37] is employed to perform electrochemical decomposition of urinal water, where the cathodic process is simulated for urea and uric acid in water with discharged ammonium ions, and the anodic process is modeled for urea and uric acid in water containing discharged hydroxide ions [38]. Previously, urinal water has been used to produce hydrogen via electrochemical oxidation with an inexpensive nickel catalyst, and it has been reported that ~36% cheaper hydrogen can be produced by using urea that requires 30–32% less thermal energy compared to water electrolysis [39]. With the limitations in simulation time and simulated domain size, the CPMD work considers 0.048 fs as a timestep for thermodynamic stability and 1 nm^3^ as a simulation cell size containing 100 atoms (solute and solvent). The simulation starts when the cations and anions discharge near the anode and cathode to produce a mixture containing only highly reactive radicals. In the anode, during the initial phases of the simulation till ~100 fs, the oxidation of urea molecules is noted with the formation of oxygen (Figure 5), and as the simulation progresses further, a ring-opening reaction is observed after 1.5 picoseconds that includes multiple dehydrogenation steps, illustrated in Figure 6. The addition of ammonia (NH_3_) and HCNO releases the urea, which gets oxidized during the reaction. In the cathode, the pure form of hydrogen is produced over a simulation time required together for 8 discharged ammonium molecules, 2 uric acid moieties, and 12 water molecules. It was also noted that catalysts and stirring can accelerate the production of thermodynamically stable products (CO_2_, H_2_O, and N_2_) in the anode [38].

**Figure 7:**
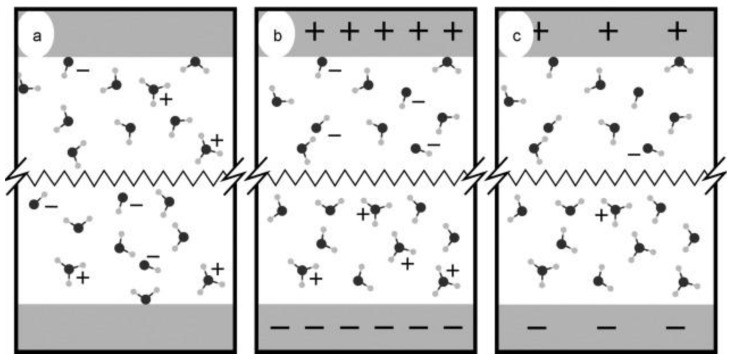
(**a**–**c**) Schematic of the electrolysis process. (**a**) The random distribution of the generated cations and anions in the water without the applied electric fields. (**b**) After applying the electric field potential, the cations (H_3_O^+^) and anions (OH^−^) move toward the negative (cathode) and positive (anode) electrode surfaces. (**c**) Electron transfer occurs, which makes the solution highly reactive, and the process continues. Reprinted with permission from [40].

Similar studies using CPMD simulations have been performed on water electrolysis till the oxygen and hydrogen are formed at the anode and cathode, respectively [40]. From these simulations at 300, 325, and 350 K temperatures, it is found that many unstable intermediate ionic compounds form during the electrolysis process, like singlet and triplet hydrogen peroxide, $H\dot{O_{2}}$, formed in an anodic reaction, while hydrogen is generated by proton transfer between water molecules. These computations have been performed in the diffusion layer or the outer Helmholtz layer from the electrode. The schematic representation of the electrolysis process is displayed in Figure 7a–c. It is reported that the first hydrogen atom forms approximately within 0.7 ps of simulation, but the second one does not emerge even after a simulation time of 8.7 ps. Nonetheless, an unstable intermediate radical $H_{13}O_{6}^{+}$ has been reported, which prevents proton transfer into the system, causing less hydrogen formation.

**Figure 8:**
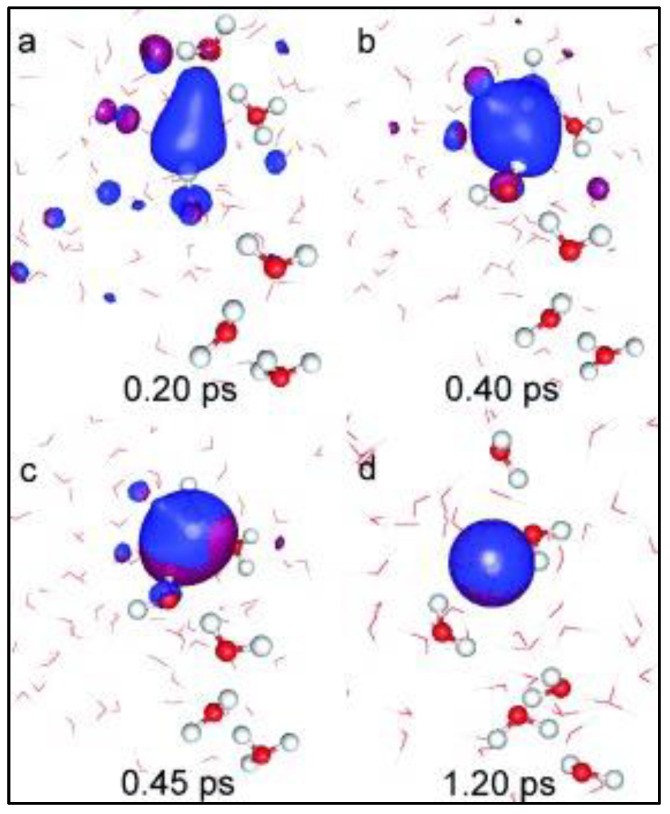
The formation of hydrogen from the ionic interaction reaction during the molecular dynamics simulation. The blue densities represent the electron (orbital) clouds, while the water molecules are presented as red–white ball–stick models. (**a**,**b**) During the initial stages of the simulation, the H_3_O^+^ ions are separated from each other, but as the simulation progresses, (**c**) the proton transfer initiates, and (**d**) the two hydrogen atoms combine to form the H-H σ bond at 1.2 ps. Reprinted with permission from [40].

**Figure 9:**
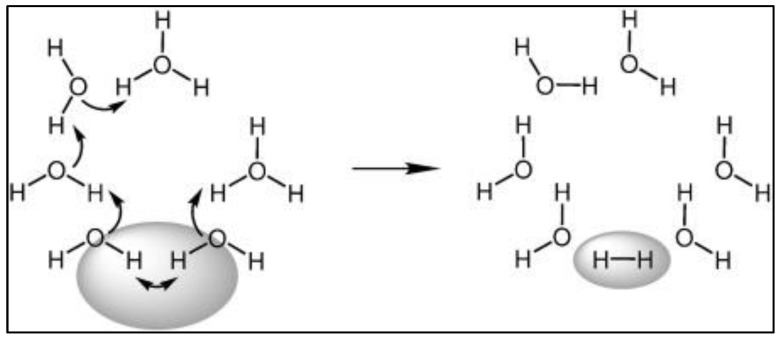
The sequence of reactions during electrolysis in an aqueous medium. Initially, the system remains neutral, while the delocalized electron cloud becomes localized after the formation of hydrogen. Reprinted with permission from [40].

The study concludes that the first hydrogen is obtained by the reaction of two moles of the unstable compound $\dot{H_{3}O}$ within 1.2 ps of simulation time, as shown in Figure 8a–d. Here, the formation of the electron clouds during the initial phases of ionic transfer is denoted by blue densities, and the formation of hydrogen is shown in Figure 8d. The chemical reaction for the hydrogen formation from intermediate compounds during this process is shown in Figure 9. It is found that the conservative cathode and anode reactions are initiated after the successful completion of electron transfer; hence, the rate-determining step is the diffusion and electron transfer, while surface modifications on the electrodes can play a vital role in determining the rate of reaction [40].

Various reactive molecular dynamics (RMD)-based studies have been used to study the kinetics of alkaline water electrolysis and inter and intramolecular water structures using different thermal and electric field potentials [41,42,43]. Lopez-Plascencia et al. applied thermal and electric fields to characterize the oxygen–oxygen, hydrogen–hydrogen, and oxygen–hydrogen bond strength during temperature-driven water splitting (thermolysis) and electric field-induced water splitting (electrolysis) [41].

**Figure 10:**
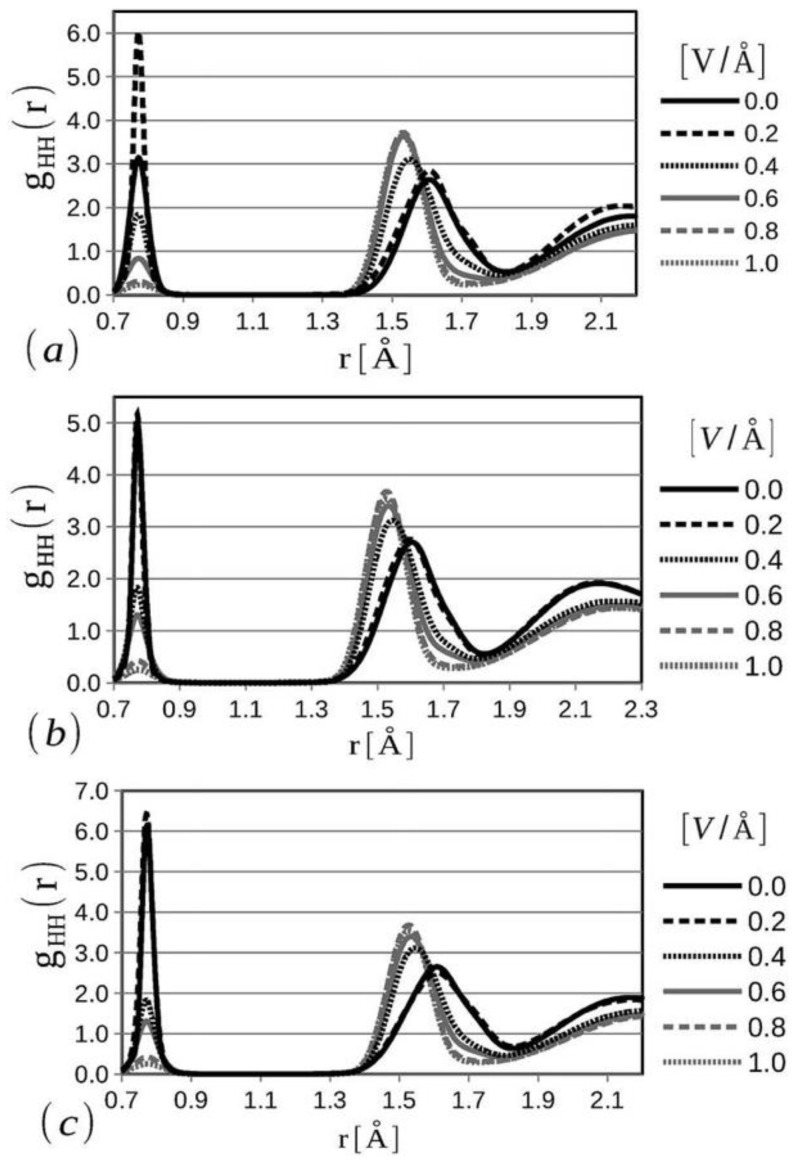
Molecular dynamics prediction of the radial distribution function of H-H bonds during water splitting under the application of external electric field potential and different temperatures, (**a**) at 280 K, (**b**) at 320 K, and (**c**) at 360 K. Reactive forcefields (ReaxFF) are applied in the simulation. Reprinted with permission from [41].

The formation of hydrogen and oxygen diatomic molecules, di-hydroxide anions, and similar molecular states have been observed at the solid and liquid phases of water from the radial distribution functions of all three bonds (O=O, H-H, and O-H) at different temperatures using varying electric field potentials in the simulation domain. H-H radial distribution function can be considered one of the most important parameters to interrogate the water electrolysis at a microscopic level when an external electric field force is applied (Figure 10) [41]. Note that the presence of the hydrogen diatomic molecule is dictated by the presence of the first peak, while with the increase in the applied electric field potential, the skewness in the geometry of water molecules increases, causing the second peak to shift to the left with a higher intensity. Also, melting-like characteristics are observed due to the applied electric field at larger radial distances, which is the primary cause for a decrease in the third peak intensity of the H-H radial distribution function at >0.4 V/Å [41]. With the increase in temperature from 280 K to 360 K, a change in the first peak can be observed at zero and lower electric fields. This study concludes that water-splitting behavior and diatomic hydrogen formation are enhanced by the application of homogeneous electric fields with values comparable to those occurring in common electrolysis experimental setups [41].

**Figure 11:**
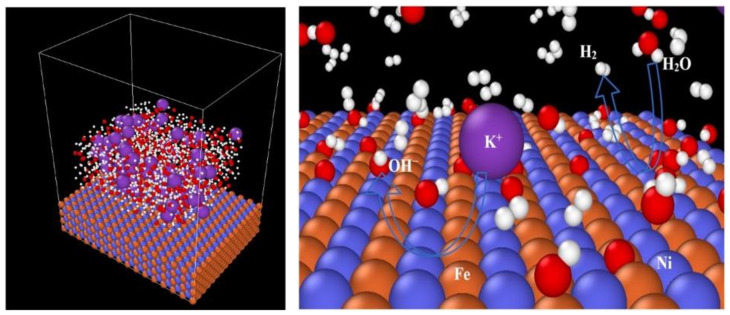
(**left**) Simulation domain for Fe/Ni heterometallic surface. (**right**) Hydrogen evolution snapshot at 0.2 ns during the simulation on an electrocatalytic surface (purple: potassium, blue: nickel, red: oxygen, white: hydrogen, and brown: iron atoms). After getting in contact with the heterometallic surface, water (H_2_O) splits into H^+^ and OH^−^ ions, which further leads to the formation of hydrogen molecules (H_2_), as shown by the arrowheads. Reprinted with permission from [43].

Similar RMD-based studies have been undertaken to demonstrate how the kinetic information of complex chemical reactions changes in an alkaline (30% KOH) environment at 300–550 K when using a heterometallic surface (NiO, Fe/Ni, and Pt/Ni) rather than monometallic surfaces (Ni and Pt) [43]. Here, a 6.0 × 4.5 × 7.5 nm^3^ periodic box is used for the hydrogen evolution reaction simulation, shown in Figure 11 (left), while Figure 11 (right) is a snapshot of the hydrogen evolution process on the electrocatalytic surface at 0.2 ns. The finding includes that systematically altering the composition of the surface by the integration of a second metal into the monometallic surfaces and aligning the ratio can dramatically increase the progressive alkaline electrolytic hydrogen evolution reaction (EHER) activity. These findings suggest that it is efficient to incorporate bimetallic component active sites for the elementary steps to promote hydrogen evolution reaction from alkaline electrolysis.

**Figure 12:**
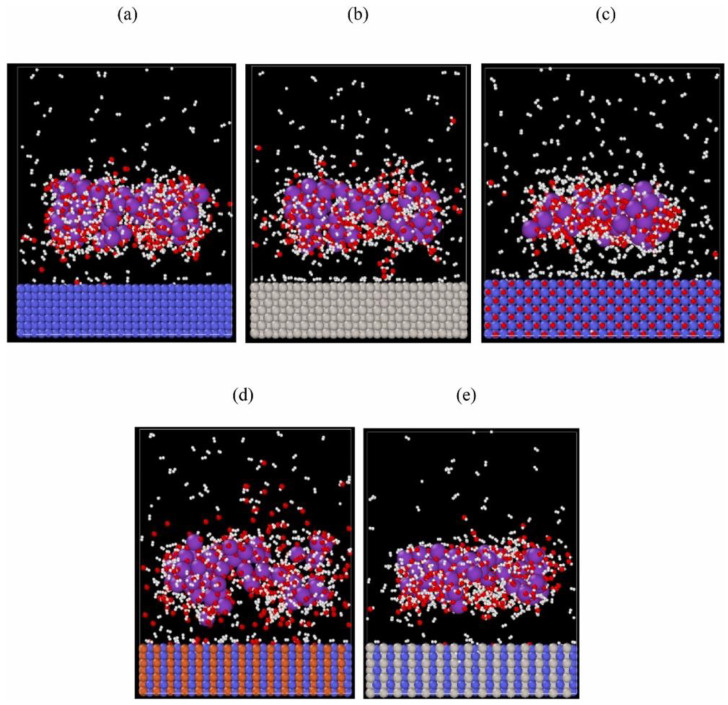
Hydrogen evolution recorded at 5 ns and 300 K on different mono and heterometallic catalytic surfaces like (**a**) Ni, (**b**) Pt, (**c**) NiO, (**d**) Fe-Ni, and (**e**) Pt-Ni (purple: potassium, blue: nickel, red: oxygen, white: hydrogen, brown: iron, and grey: platinum atoms). Reprinted with permission from [43].

RMD results also show that the activation energy of NiO-based surfaces is higher than Ni and Pt monometallic surfaces for the dissociation of H_2_O into hydrogen and oxygen; thus, it is easier to form hydrogen on a Ni or Pt surface than on a NiO surface. The systematic alteration of the composition of the mono-metallic surface into a heterometallic surface enhances the ability to combine specific active sites for different elemental reactions to improve the alkaline EHER and find the optimal overall reaction. The snapshot of the predicted hydrogen evolution reaction for the temperature range of 300–550 K on different catalytic surfaces at 5 ns is presented in Figure 12. The overall results show that across all temperatures, the hydrogen evolution rate of the five catalysts follows the trend of FeNi > Ni > Pt > PtNi > NiO, implying that the FeNi heterometallic surface is capable of generating H_2_ molecule with high evolution rates [43]. Oyinbo et al. performed similar RMD simulations to compare nickel-based heterometallic catalysts in an alkaline potassium hydroxide (KOH) solution for H_2_ generation using ReaxFF (reactive force field) potential [42].

**Figure 13:**
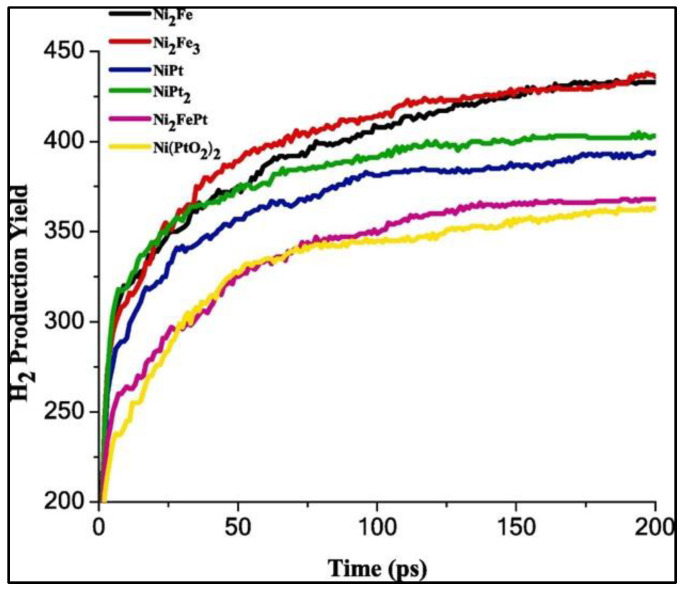
The time rate of hydrogen production yield found in an alkaline KOH (30%) solution with Ni_2_Fe (χ_Ni_ = 67%, χ_Fe_ = 33%), Ni_2_Fe_3_ (χ_Ni_ = 40%, χ_Fe_ = 60%), NiPt (χ_Ni_ = 50%, χ_Pt_ = 50%), NiPt_2_ (χ_Ni_ = 30%, χ_Pt_ = 70%), Ni_2_FePt (χ_Ni_ = 50%, χ_Fe_ = 25%, χ_Pt_ = 25%), and Ni(PtO_2_)_2_ catalysts at 298 K. Reprinted with permission from [42].

The significance of the transitional metals (Ni, Fe, and Pt) and their oxides have been explored for the catalytic efficacy of the Ni-based catalyst for H_2_ evolution. Ni-Fe and Ni-Pt induce major promoting effects on the Ni-based catalyst, with an improvement in the hydrogen generation rate relative to the Ni-Fe-Pt heterometallic catalyst. On the other hand, only a marginal improvement in the catalytic performance of the Ni-based catalyst is noted with Ni-Fe-O and Ni-Pt-O catalysts. These analyses corroborate that the efficiency of alloy catalysts for H_2_ generation decreases in the following order: Ni_2_Fe_3_ > Ni_2_Fe > NiPt_2_ > NiPt > Ni_2_FePt > Ni(PtO_2_)_2_, according to their hydrogen production yield and activation energies at different temperatures (Figure 13). These results assert that regardless of the concentration of added transition metals like Fe and Pt, the electrocatalytic efficiency of Ni-based catalysts increases. The maximum hydrogen evolution rate (R_max_) and Gibbs free energy at 298 K (ΔG_298_) are listed in Table 1 [42].

**Figure 14:**
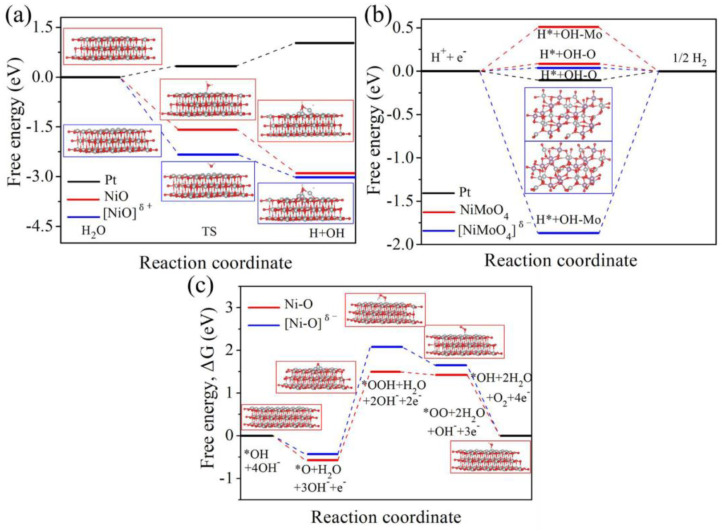
(**a**) Free energy barrier of water dissociation on Pt, NiO, and [NiO]^δ+^ surfaces at initial, transitional, and final states. (**b**) Gibbs free energy change for HER on Pt, NiMoO_4,_ and [NiMoO_4_]^δ+^ surfaces, and (**c**) Gibbs free energy change for HER on Ni-O and [Ni-O]^δ−^ surfaces. Reprinted with permission from [49].

Besides all the Ni-based hetero-catalysts that demonstrate potential in reducing the overpotential of the HER and eventually generating higher hydrogen yield in alkaline solutions, experimental and computational studies reveal that Mo-based catalysts are efficient in catalyzing the HER and bifunctional Ni-Mo compounds can be treated as promising sources for catalyzing both the OER and HER [44,45]. Other than transition metals, the Ni-Mo solid solution nanowire array electrodes with 1.6% Mo exhibit significantly good performance in attaining extremely low overpotentials (~17 mV at 10 mA/cm^2^) with an onset potential as low as 3 mV, comparable to the commercially available Pt/C catalysts and superior to most of the state-of-the-art Pt-free catalysts in alkaline medium [46]. An important factor when considering the electrocatalytic effect of Ni-Mo electrodes is the role of the support materials. The highest current is reported for Ni-Mo electrocatalysts with Cu-based support material, followed by Ni and Ti-based ones [47]. A study on vertical graphene-supported Ni-Mo electrodes showed impressive performance in an alkaline medium with a low HER overpotential of 70.95 mV at 10 mA/cm^2^, superior to the performance of other substrate-based Ni-Mo electrodes for the same applications under similar operating conditions [48]. Also, seawater splitting is gaining attention, especially for its abundance, but there exist some challenges like chlorine evolution reaction on the anode, which can dominate over the OER and further corrode the catalyst and the substrate. An experimental–computational study based on a nickel foam-supported Ni-Mo hybrid catalyst revealed excellent catalytic behavior and corrosion resistance in seawater splitting, where a cell voltage of 1.563 V is used to attain a high current density of 10 mA/cm^2^ [49]. Their computational results using density functional theory (DFT) contribute to understanding the electronic structure of NiMoO_4_ and NiO on their performance in the OER and HER. Figure 14 illustrates the free energy barriers with reaction coordinates for the HER and OER under different electrocatalysts as computed from the DFT calculations. Figure 14a indicates that [NiO]^δ+^ promotes faster water dissociation than normal [NiO] and Pt electrodes, which further enhances the formation of adsorbed hydrogen. Figure 14b,c show that [NiMoO_4_]^δ−^ is the most efficient catalyst for hydrogen production in the HER using seawater splitting, and Ni-O achieves a better OER process as suggested by its low free energy barrier compared to [NiO]^δ−^ (where δ+ represents the electron deficiency and δ− represents the electron excess) [49]. Table 2 lists the performance characteristics of the HER of different available electrocatalysts.

## 4. Computational Models of Molecular Hydrogen Storage

Computational studies have been used to predict how different materials store hydrogen under varying conditions. Ogawa et al. performed first principle calculations and classical molecular dynamics (MD) studies to determine how hydrogen in a gas phase gets stored in model BCC metallic nanoparticles as a function of the length and energy of metallic H bonds [59]. Furthermore, MD simulations have been performed to analyze hydrogen adsorption capabilities and the diffusion of atomic and molecular hydrogen on graphene and graphite nanostructures [60,61,62].

**Figure 15:**
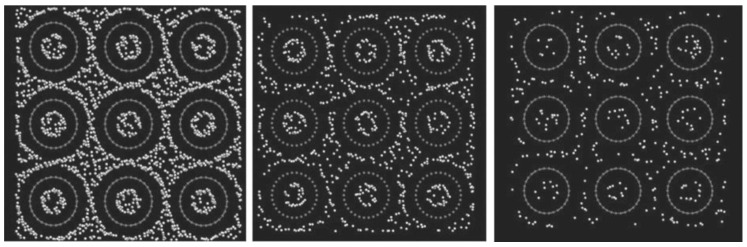
Hydrogen storage in carbon nanotube bundles. Snapshots from grand canonical Monte Carlo simulations under 100 bar pressure at 77 K (**left**), 175 K (**middle**), and 293 K (**right**). Reprinted with permission from [35].

Figure 15 reproduces results obtained from grand canonical Monte Carlo simulations that show how carbon nanotubes can store hydrogen, but only the cryogenic conditions render the highest hydrogen storage capacity. Classical MD simulations predict that there is an optimized bond length for which the absorption of hydrogen gas is maximum, but with any increase/decrease in bond length, the storage capability decreases. Longer H bonds prevent the migration of H atoms from the surface, but for short H bonds, H atoms stationed at the interstitial gap have more potential energy than for the optimized bond length. The computational model is also applicable to hydrogen storage in nanoclusters [59]. A homogeneous surface layer on the nanoparticles is generated for strong metal H bonds, while an inhomogeneous distribution of hydrogen gas inside nanoparticles is attributed to the sluggish diffusion of hydrogen and lattice distortion due to hydrogen absorption. MD simulations predict that pressure, temperature, number of layers, and inter-layer spacing for multi-layer graphene play an important role in maximizing hydrogen adsorption as characterized by binding energy, binding force, and gravimetric hydrogen storage capacity (HSC) [60]. A greater amount of hydrogen adsorption or larger density of hydrogen layers on a single-layer graphene sheet (i.e., higher HSC) is obtained at a correspondingly higher pressure (15 MPa) and lower temperature (77 K), and the dependence of HSC on temperature is higher compared to the pressure because at a considerably higher temperature, the kinetic energy of hydrogen molecules increases, and they start to desorb from the graphene layers. In a prior study that considers four different interlayer spacings of 0.35, 0.70, 1.05, and 1.40 nm, it is found that when four layers of graphene are used with an interlayer spacing of 0.7 mm, the HSC increases by 11.15 wt.% compared to a single layer [60]. Also, with an interlayer spacing of 1.40 nm, the gravimetric HSC increases 4.21 times that for the 0.35 nm spacing, but the increase is rather limited when the spacing is >1.40 nm because of the higher absorbable and storage area.

Similar studies on the diffusion of hydrogen atoms through isolated graphene sheets have been performed using path-integrated molecular dynamics (PIMD) simulations and transition state theory to examine the effect of impurity mass on the properties of hydrogenic point defects, which is the chemisorption of hydrogen and deuterium on a single layer of the graphene sheet [61]. The finite-temperature properties of these point defects have been analyzed in the range from 200 to 1500 K with a tight-binding potential fitted to density-functional calculations for vibrational properties, including the contributions of anharmonic effects. Although the predicted results show an increase in diffusion for both hydrogen and deuterium atoms, hydrogen adsorbed on graphene cannot be accurately described as a particle moving in a harmonic potential. Likewise, MD simulations based on the NVE ensemble for a graphite supercell containing 64 C atoms and one impurity (H or H_2_) atom are performed to calculate hydrogen diffusion coefficients at a higher temperature [62]. Two graphite sheets, each a 4 × 4 graphene supercell of size 4a = 9.84 Å with an average distance between sheets of 3.35 Å, are employed to characterize hydrogen adsorption in the interlayer of the graphite structure. The results show that the relaxation of the C atoms in the nearest graphite layers augments the hydrogen diffusion, with the diffusion coefficient of molecular hydrogen being one degree higher than that of the hydrogen atom at 1000 K and almost four orders more at 300 K.

The storage efficiency has been predicted for graphene bubble structures that have been used to accumulate hydrogen at 300 K and 1 bar [63]. NPT ensemble-based MD simulations are carried out where the effects of 2–5 graphene layers, their density, and their bubble sizes are investigated, and the results show that at ambient conditions, the highest volumetric and gravimetric hydrogen storage efficiency of ~45 kg/m^3^ and ~3.75 wt.% are achieved. With an increase in the number of graphene layers, the hydrogen storage amount decreases marginally, but the gravimetric density drops rather sharply [60,63]. Therefore, these studies reflect that the maximum hydrogen storage efficiency can be improved at high pressure, low temperature, and large graphene interlayer spacing. The storage of molecular hydrogen in bubbles is a more efficient method under ambient conditions. The storage capacity of hydrogen has been analyzed in a novel 3D carbon structure-pillared graphene bubble system made of semi-ellipsoidal graphene bubbles having different sizes [64].

**Figure 16:**
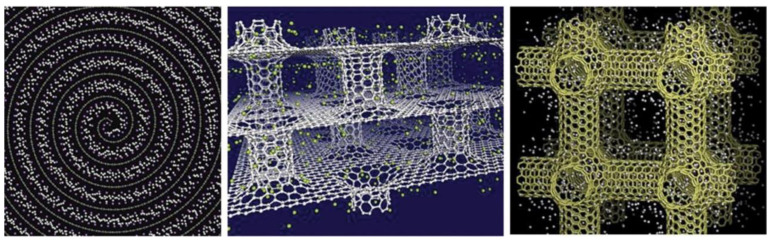
Hydrogen storage in three different types of carbon-based nano-architectures: nanoscrolls (**left**), pillared graphene (**middle**), and porous nanotube network (**right**). Snapshots are from grand canonical Monte Carlo simulations. Reprinted with permission from [35].

**Figure 17:**
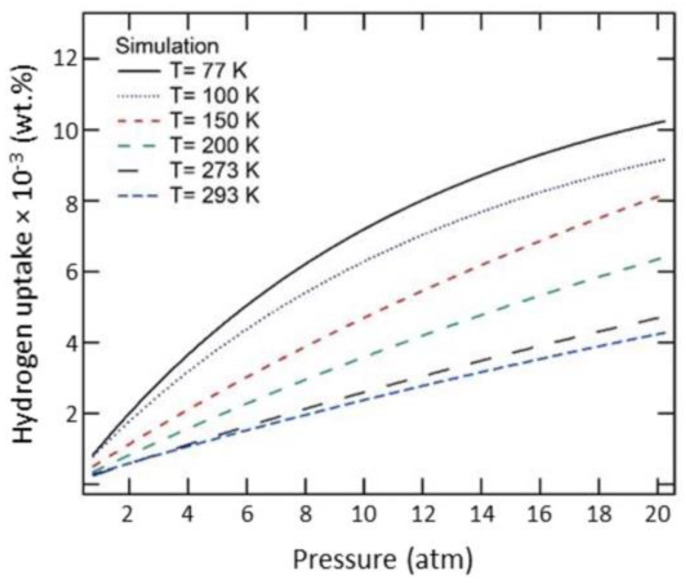
Hydrogen adsorption capacity with varying operating pressures for graphene nanofibers at different temperatures. Reprinted with permission from [65].

Figure 16 represents the storage of hydrogen in different types of porous carbonaceous structures. Hydrogen storage properties and the internal pressures of pillared graphene bubble structures at different pressures, temperatures, and interlayer spacings from MD simulations [64] showed that the storage capacity in the pillared graphene bubble structures can be maximized by decreasing the temperature and increasing the pressure and the graphene interlayer spacing. The MD simulations demonstrate that the maximum gravimetric and volumetric H_2_ densities inside the system are 13.7 wt.% and 121.6 kg/m^3^, respectively, at 77 K and 100 bar, and the values of these for a developed system are 21.3 wt.% and 210.3 kg/m^3^ [64]. Therefore, it can be concluded that pillared graphene bubble structures have a higher potential for hydrogen storage. MD simulations have been carried out to calculate the hydrogen uptake at different temperatures from 77 K to 298 K in ZSM5, graphite nanofiber, graphene oxide framework, and reduced graphene oxide to compare and verify the measurements from previous experiments. At 77 K, the simulation predictions agree partially with the experimental results, but it does not match at higher pressures [65]. The hydrogen intake capacity decreases with an increase in temperature for all these materials. Figure 17 replicates this interpretation wherein the hydrogen uptake capacity increases with the decrease in temperature. At higher temperatures, the uptake capacity varies linearly with increasing pressure, whereas below 100 K, the hydrogen uptake capacity attains a saturation for pressure > 20 atm [65].

**Figure 18:**
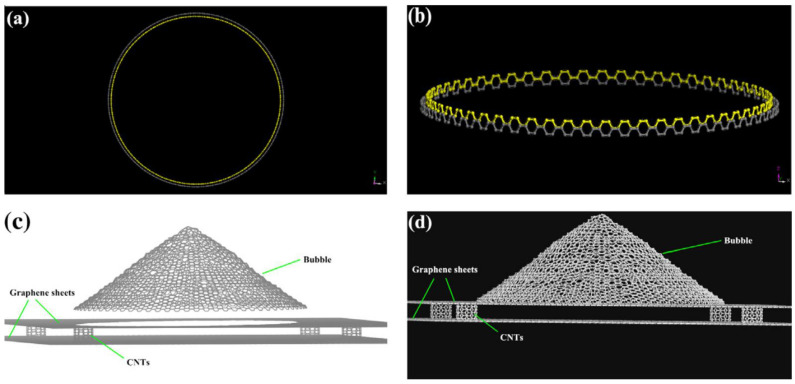
(**a**) Vertical and (**b**) lateral views of the configuration of CNTs. (**c**) Graphene bubbles, graphene sheets, and (6, 6) CNTs prepared to form a building block. (**d**) Schematic of the 3D pillared graphene bubble structure, which is the initial structure optimized by MD simulations. Reprinted with permission from [64].

**Figure 19:**
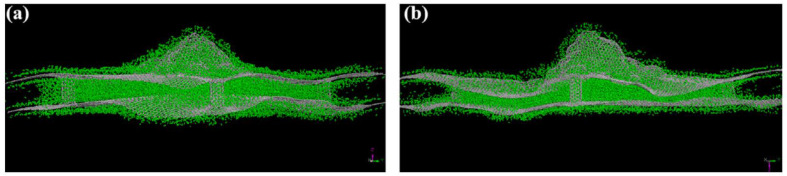
Pictorial representation of hydrogen storage in pillared bubble graphene structures (**a**) α and (**b**) β at 77 K and a pressure of 100 bar. Reprinted with permission from [64].

Figure 18 reproduces the carbon nanotube (CNT) connections and the graphene sheets and 3D pillared graphene bubbles, while a pictorial representation of hydrogen storage in the pillared graphene bubble structure, when the outer surface adsorption is considered, is displayed in Figure 19. Georgakis et al. performed several MD simulations to examine the physical adsorption of molecular hydrogen on carbonaceous structures using three different models [66], viz., the single-sheet model (SSM) having only two parallel sheets, the inner holes model (IHM), and the hollow walls model (HWM) comprising structural imperfections in the form of pits and holes in their structure. In all three models, it is found that hydrogen density is higher than the corresponding density for liquid hydrogen, and the addition of extra sheets to the walls does not result in any enhancement of the hydrogen adsorption capacity. The SSM exhibits the best results for the % w/w hydrogen adsorption (~18.53% being highest) relative to the IHM (~6.33% being highest) and the HWM (~6.91% being highest) [66]. Furthermore, these results suggest that the design of a targeted material for hydrogen storage must be directed towards manufacturing a lightweight material with a slit-shaped pore (a combination of the SSM and the HWM) to increase the adsorption and augmentative effect of the holes of the carbonaceous sheet.

Research efforts to predict the effect of hydrogen gas and its storage capacity on materials other than carbonaceous graphite or graphene layers include the storage capability of hydrogen gas by the clathrate of hydroquinone (HQ) and hydrogen adsorption by silica [67,68]. Rodriguez et al. used quantum mechanical calculations to define the structure of HQ at an atomic level, followed by MD simulations to model the HQ clathrate during the successive processes of capture and release of hydrogen and its diffusion inside the HQ clathrate structure [67]. The diffusion of H_2_ inside the HQ lattice contributes to the occurrence of spontaneous double occupation per void. The results indicate that the potential use of HQ clathrate for H_2_ storage is advantageous in the high volumetric capacity and at atmospheric pressures, as well as the mild thermodynamic conditions involved in the processes of capture and release. Similarly, MD simulations have been used for qualitative analysis of the effect of pressure on the hydrogen adsorption on silica [68]. The simulations performed at a temperature of 273 K and by varying pressure from 1 atm to 10 atm show that the amount of hydrogen molecules adsorbed at 10 atm pressure is > 10× higher than the amount adsorbed at 1 atm, i.e., hydrogen adsorption is highly pressure-dependent.

**Figure 20:**
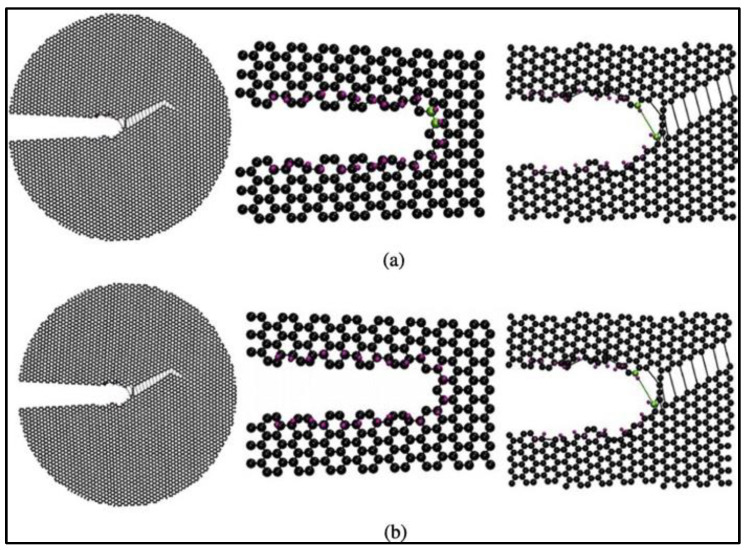
Armchair-type crack propagation due to the adsorption of out-of-plane hydrogen atoms under mode I loading condition in graphene when hydrogen atoms are adsorbed on (**a**) only one crack surface and (**b**) both the crack surfaces at the crack tip. Carbon and hydrogen atoms are shown in black and purple, and the broken bond at the crack tip is shown in green. Reprinted with permission from [69].

**Figure 21:**
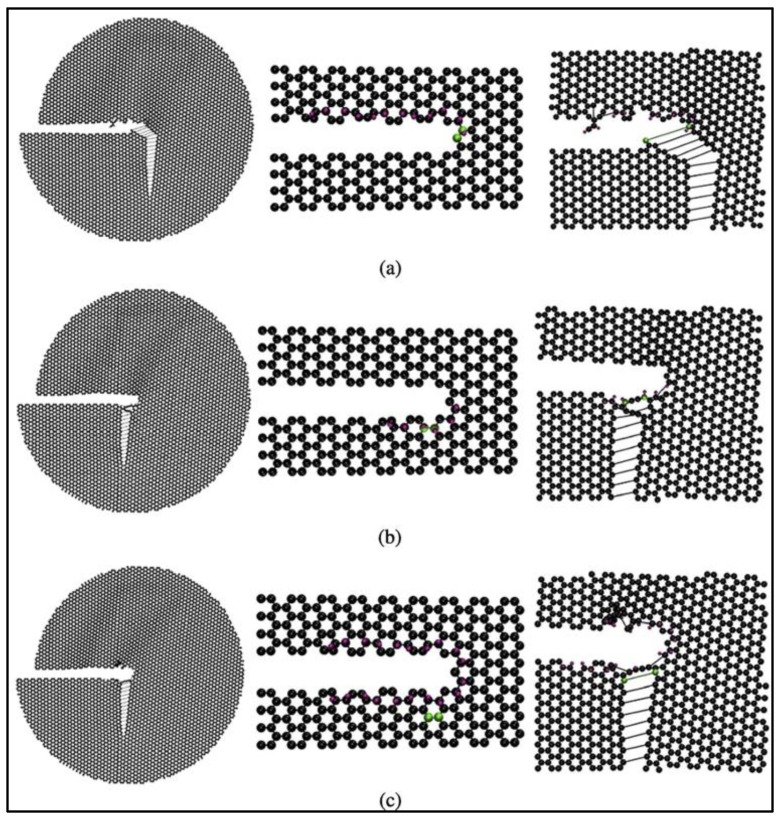
Armchair-type crack propagation due to the adsorption of out-of-plane hydrogen atoms under mode II loading condition in graphene when hydrogen atoms are adsorbed on (**a**) only the top surface, (**b**) both top and bottom surface, and (**c**) only the bottom surface at the crack tip. Carbon and hydrogen atoms are shown in black and purple, and the broken bond at the crack tip is shown in green. Reprinted with permission from [69].

The effects of hydrogen adsorption on the fracture mechanics of graphene have also been investigated [69]. The simulations predict that the adsorption of hydrogen atoms on the crack tip or surface leads to a reduction in graphene toughness and can alter the crack propagation paths. Based on the results of these MD simulations, the locations of carbon atoms to which hydrogen can attach are the predominant factor in the degree of impact that hydrogen atoms have on the fracture properties of graphene. The in-plane hydrogen atoms do not affect the crack surface or tip morphology and do not impact the crack propagation paths. On the other hand, the adsorption of out-of-plane hydrogen atoms leads to out-of-plane deformation of carbon atoms, causing the crack tip and edges to be distorted. The out-of-plane hydrogen atoms reduce the critical stress intensity factors of graphene sheets significantly [69]. Lastly, the influence of out-of-plane hydrogen atoms on armchair cracks is the most profound among all the cases studied. Figure 20 presents the armchair crack propagation caused by the out-of-plane hydrogen atom under mode I fracture loading (where fracture happens in a plane perpendicular to the direction of applied normal force), and Figure 21 replicates the same under mode II fracture loading conditions (where fracture happens in a plane parallel to the direction of the applied shear force). In mode I, hydrogen atoms are considered to be attached to one or both the crack surfaces, while in mode II, hydrogen atoms are assumed to be attached to the top, bottom, or both surfaces of the crack.

## 5. Outlook

In summary, we analyzed the current state of the art in the computational materials and process modeling of hydrogen production and hydrogen storage at a molecular scale. In particular, all-atom molecular dynamics (MD) simulations have been widely used to identify appropriate electrochemical catalysts for the hydrogen evolution reaction (HER) from water-based electrolysis. Likewise, MD simulations have been implemented to understand the hydrogen storage capacity and storage efficiency of a wide variety of materials, including porous nanocrystalline structures. For hydrogen evolution to initiate from water electrolysis, specified electrocatalytic surfaces are essential, with the heterometallic Ni-based electrocatalysts being widely used because they increase the hydrogen evolution rate. Similarly, for hydrogen storage, porous nanostructured materials exhibit higher molecular hydrogen storage capacity, where the storage efficiency increases with the increase in storage pressure and decreases with storage temperature for the majority of the materials. The computational tools to predict the hydrogen production capability and the storage efficiency at different pressures, temperatures, and environmental conditions streamline the processes but require the use of suitable interaction potentials to mimic experimental measurements.

It is widely reported in the literature that most of the current hydrogen production employs either alkaline water electrolysis or methene-based natural gas reforming processes. For the hydrogen generation reactions using electrolysis, computational efforts are predominant as applied to alkaline water electrolysis with various electrocatalysts. To determine the effectiveness and optimize hydrogen production processes, predictive modeling can significantly guide and streamline the physical experiments. To determine the optimal alkaline concentration under targeted thermodynamic conditions for enhanced hydrogen evolution, ab initio molecular dynamics (AIMD) and Car–Parrinello Molecular Dynamics (CPMD) are the emergent tools. Likewise, to compare different aqueous alkaline solutions beyond KOH, novel Ni and Pt-based electrodes can enable examining the hydrogen evolution efficiencies. Notably, the importance of monometallic and heterometallic surfaces and the significance of the transitional metals (Ni, Fe, and Pt) and their oxides on the catalytic efficacy of the H_2_ evolution have been explored widely in the literature [41,42,43]. Hydrogen from urinal water and ethanol–water mixer with a gradual increase in field intensity indicates that a certain field intensity is needed before the dissociation initiates, also known as the dissociation threshold electric field intensity. Likewise, the electron transfer process from a surface electrode and the use of accurate interatomic charge distributions are crucial for electrolysis under an external electric field. Thus, the application of electric fields as an alternative for electrocatalysts requires extensive examination.

During electrolysis, hydrogen evolves at the cathode, and in consequence, the impact of hydrogen on the cathodic material is critical because hydrogen exhibits strong diffusion and adsorption characteristics at elevated temperatures and pressures [40,68]. Hydrogen has the potential to initiate the embrittlement of metal alloys, endangering the electrode materials [70]. In the realm of hydrogen storage, typically carbonaceous materials such as graphene and graphite stacks have been used to predict hydrogen storage capacity and efficiency under ambient thermophysical conditions. Also, the hydrogen retention rate dictates how well hydrogen (mostly in gaseous form) remains bound within the interstitial gaps of the porous (carbonaceous) material structures. One of the growing global technological needs is to store hydrogen effectively with a higher hydrogen retention rate and storage capacity. Carbon nanotube (CNT)-based composites show significant hydrogen storage capacity under ambient conditions and as low as 77 K [71]. Parametric studies on these CNT-based composites reveal their dependence on temperature, diameter, and volume fraction that can further impact their hydrogen storage abilities [71,72,73]. To address the related material challenges, deeper scrutiny of hydrogen storage capacity and hydrogen retention rate at elevated temperatures (>350 K) for materials beyond graphitic structures, such as CNTs, across different pressures and crystal structures is imminent.

Lastly, MD simulations and DFT-based computational studies can effectively predict the dissociation and recombination time of hydrogen molecules within a porous structure. The efforts can be extended to systematic studies of graphene bubble structures accumulating H_2_ at different temperatures, pressures, and interlayer chemistries. Molecular simulations are an important tool to predict and qualitatively analyze hydrogen production and its impact on hydrogen storage for different material systems. By simulating the trajectories, interactions, and reactions of hydrogen in different environments, MD simulations reveal valuable information about diffusion mechanisms, adsorption energies, and reaction pathways. DFT calculations, on the other hand, offer a quantum chemical perspective on the electronic structure and energetics of the materials and provide insights into the binding energies of hydrogen with various elements, aiding in the potential discovery of novel materials for hydrogen storage and catalysis. The integration of MD, DFT, and machine learning techniques can enable a holistic understanding of hydrogen–material interactions. For instance, MD simulations and DFT calculations can provide training data at different material lengths and time scales that serve as the input for machine learning algorithms to identify key descriptors for materials with exceptional hydrogen adsorption or dissociation properties. This iterative process allows for the refinement of both simulations and machine learning models, resulting in a comprehensive understanding of the mechanisms underlying hydrogen-related phenomena. The synergistic use of computational simulations and machine learning has profound implications for both theoretical and practical advancements in hydrogen production and storage. By guiding the experimental efforts toward materials with desired properties, an integrated computational paradigm accelerates the discovery of efficient catalysts, adsorbents, and storage materials. Moreover, the fundamental knowledge gained from simulations contributes to the design of novel materials tailored for hydrogen production, storage, and transport. Continued research adopting these computational engines will facilitate progress in clean hydrogen production and mitigate the next-generation challenges in hydrogen storage. The synergy between computational simulations and experimental measurements has the potential to enhance our understanding of the underlying processes and fast-track the transition toward a hydrogen-based energy economy.

## Figures and Tables

**Table 1:** Maximum hydrogen evolution rate (R_max_) and Gibbs free energy (ΔG) at 298 K from RMD simulations. Reprinted with permission from [42].

Catalysts	R_max_ (mol/ps)	ΔG_298_ (kJ/mol)
Ni	2.034	38.272
Pt	1.984	37.370
Ni_2_Fe	2.165	39.365
Ni_2_Fe_3_	2.180	38.156
NiPt	1.940	38.001
NiPt_2_	2.015	37.557
Ni_2_FePt	1.840	33.513
Ni(PtO_2_)_2_	1.815	35.771

**Table 2:** Comparison of performance characteristics of various electrocatalysts for their HER under different conditions and solutions.

Electrocatalysts	Overpotential Voltage (mV)	Current Density (mA/cm^2^)	Tafel Plots (mV/dec)	Electrolyte
CF (bare Cu-foam) [50]	570	100	152	1 M KOH
CoWO_4_/CF [50]	237	100	125	1 M KOH
CoMnO_4_/CF [50]	198	100	131	1 M KOH
W-CoMnO_4_/CF [50]	102	100	117	1 M KOH
MoS_2_/CC (Carbon cloth) [51]	210	100	107	1 M KOH + 0.4 M N_2_H_4_
Co(OH)_2_/CC [51]	265	100	131	1 M KOH + 0.4 M N_2_H_4_
Co(OH)_2_/MoS_2_/CC [51]	134	100	88	1 M KOH + 0.4 M N_2_H_4_
Ni-Cu-P@Ni-Cu [52]	590	100	63	1 M KOH + 0.5 M N_2_H_4_
NiS_2_/TiM [53]	484	100	22	1 M KOH + 0.3 M N_2_H_4_
NiSe/MoSe_2_/CC [54]	106	10	14	1 M KOH
NiSe/CC [54]	-	10	25	1 M KOH
NiMoO_4_/CC [54]	450	10	136	1 M KOH
MoSe_2_/CC [54]	237	10	372	1 M KOH
NiMo-65 (5% Mo Salt) [46]	17	10	28	1 M KOH
Ni_4_Mo nanosheet [55]	35	10	45	1 M KOH
NiMoO_4_ [55]	288	10	142	1 M KOH
Ni-foam (NF) [55]	335	10	159	1 M KOH
Ni_2_P [56]	137	10	49	0.5 M H_2_SO_4_
Ni_5_P_4_ [56]	118	10	42	0.5 M H_2_SO_4_
CoMoP@C [57]	41	10	50	0.5 M H_2_SO_4_
Ni(Cu)/NF [58]	27	10	33	1 M KOH
Pt/C [58]	23	10	25	1 M KOH
NiMo@VG [48]	71	10	87	1 M KOH
NiMo-ammonia deposited [49]	37	10	34	1 M KOH
NiMo-ammonia deposited [49]	32	10	33	1 M KOH + 0.5 M NaCl

## Data Availability

The data presented in this study are available upon request to the corresponding author.
